# Beyond Social Media: Curated Monitoring Footage as a Biodiversity Information Source for Conservation

**DOI:** 10.3390/ani16101427

**Published:** 2026-05-07

**Authors:** Xue Yang, Chen Yang, Farui Zhang, Weichao Zheng, Tianpei Guan

**Affiliations:** 1 College of Grassland Resources, Southwest Minzu University, Chengdu 610225, China; yangxueissnow@163.com (X.Y.); yangchen@swun.edu.cn (C.Y.); zfr13258288871@163.com (F.Z.); 2 Sichuan Academy of Giant Panda, Chengdu 610081, China; 3 Sichuan Provincial Forest and Grassland Key Laboratory of Alpine Grassland Conservation and Utilization of Qinghai-Tibetan Plateau, Chengdu 610225, China

**Keywords:** mass media, conservation awareness, biodiversity data, distribution patterns

## Abstract

Timely and reliable information on species occurrence and distribution is essential for biodiversity conservation, but conventional surveys are often limited by incomplete spatial coverage, high costs, and infrequent updates. These limitations are particularly relevant for medium- and large-sized mammals, whose distributions may respond rapidly to human disturbance and land-use change. Here, we evaluated the potential of long-term, curated mass-media records to support biodiversity knowledge by analyzing The Eyes of the Secret Land (TESL), a Chinese television program based mainly on infrared camera-trap footage collected across protected areas in China. We extracted mammal records from 1829 episodes broadcast between 2019 and 2024 and compared media-exposed species richness patterns with official provincial biodiversity data using Spearman rank correlation. TESL documented 118 mammal species, including 42 nationally protected and 52 IUCN-threatened species. Media-exposed species richness was positively correlated with official richness at the provincial level (ρ = 0.68, *p* < 0.001). Generalized linear models identified episode count and latitude as significant predictors of media-exposed species richness. Comparison with IUCN range maps also identified three potential new distribution records. These results demonstrate that curated monitoring footage from a mass-media program can recover broad-scale biodiversity patterns, although inherent taxonomic and spatial biases necessitate cautious interpretation. We propose that curated monitoring footage represents a promising but underutilized supplementary data source for biodiversity assessment and conservation science communication.

## Simple Summary

Biodiversity conservation depends on knowing where animals live, but traditional wildlife surveys are expensive and cover only small areas. We explored whether a popular Chinese television program, The Eyes of the Secret Land (TESL), which films wildlife using camera traps in protected areas across China, could provide useful information about mammal diversity. By watching 1829 episodes broadcast between 2019 and 2024, we identified 118 mammal species, including many that are nationally protected or threatened. The patterns of species richness recorded by the program matched well with official biodiversity data, particularly in the species-rich southwest of China. Although the program naturally favors large, eye-catching animals, our results show that carefully curated television footage can serve as a valuable supplement to scientific monitoring—and that such programs may play an important role in both documenting and communicating biodiversity to the public.

## 1. Introduction

Reliable and up-to-date information on species distributions is fundamental for biodiversity conservation, yet conventional wildlife surveys remain expensive, logistically challenging, and uneven in spatial coverage [1,2,3]. This information gap is particularly acute for mammals, many of which are nocturnal, elusive, or inhabit remote areas that are difficult to survey systematically [4,5]. In recent years, alternative data sources—including social media records, citizen science platforms, and environmental DNA—have emerged as potentially valuable complements to traditional monitoring [6,7,8]. However, each of these approaches carries inherent limitations in terms of taxonomic accuracy, geographic bias, or temporal consistency.

The rapid expansion of digital technologies has created unprecedented opportunities to supplement conventional biodiversity data [7]. Cameras embedded in smartphones, vehicles, drones, and other monitoring devices now generate massive volumes of visual records that may contain wildlife occurrence information [8]. Accordingly, an increasing body of research has explored media-derived biodiversity data, including citizen science observations, social media content, and other digital traces of human–nature interactions [9,10,11,12]. These sources have shown substantial potential to complement traditional biodiversity datasets, particularly across broad spatial and temporal scales [13]. However, such data are also shaped by strong biases related to observer behavior, accessibility, and species detectability. Therefore, before they are used for large-scale ecological inference, their concordance with established biodiversity patterns must be critically evaluated [14,15].

Curated monitoring footage occupies a distinct position within this expanding landscape of alternative biodiversity data sources. Unlike social media records, which are generated through decentralized and opportunistic public contributions and are often shaped by recreational preferences and accessibility biases [9,13], curated monitoring footage originates from systematic field deployments conducted by trained personnel within protected areas. Similarly, while citizen science programs such as eBird (v. 3.3.5) and iNaturalist (v. 1.30.15) rely on voluntary observations that vary widely in taxonomic expertise and geographic coverage [13,14,15], curated footage is often collected using standardized camera-trap protocols and subsequently reviewed by professional editors and taxonomic experts. Although the underlying footage may originate from systematic monitoring, the curated outputs are still subject to editorial selection. This combination of systematic data collection and professional quality control gives curated monitoring footage distinct advantages: it may retain broad spatial and temporal coverage, while offering a higher degree of taxonomic reliability and ecological context than typically found in opportunistic records. Despite these potential advantages, curated monitoring footage has received little attention as a biodiversity data source, and its capacity to reflect established biodiversity patterns remains largely untested.

Among visual data sources, camera-trap records are especially important because they differ fundamentally from opportunistic public observations. Camera traps are now among the most widely used tools for mammal monitoring in protected areas and play an irreplaceable role in biodiversity assessment [16,17]. Their records are typically generated through systematic field deployment and provide direct, verifiable evidence of species occurrence, often capturing rare species and elusive behaviors in natural habitats [18]. As a result, camera-trap programs have accumulated vast quantities of video footage that are valuable not only for scientific research, but also for conservation education and public communication [19,20]. Yet beyond their use in published local studies, these archives remain underutilized, and little attention has been paid to whether curated outputs derived from such footage can also serve as informative sources for biodiversity assessment at a broader scale.

This question is particularly relevant in China, one of the world’s megadiverse countries. China spans strong biogeographic gradients and harbors highly diverse mammalian assemblages [21,22,23]. At the same time, biodiversity surveys in China remain spatially uneven [24], creating a need for complementary data sources that can broaden coverage and public engagement. In this context, “The Eyes of the Secret Land” (TESL), a television program launched in 2019, offers a distinctive case. TESL features wildlife clips collected over more than five years from infrared camera traps and other field monitoring equipment across a wide range of ecosystems and protected areas in China. Unlike social media platforms, which are dominated by decentralized and opportunistic contributions [13], TESL represents a form of curated mass media built from long-term field monitoring records. Such a dataset may simultaneously communicate biodiversity to the public and preserve ecologically meaningful information. However, whether this type of curated mass-media product can reflect broad-scale biodiversity patterns and provide useful conservation signals remains largely untested.

In this study, we extracted mammal-related biodiversity information from TESL to assess the potential of long-term, curated mass-media records as a source of biodiversity knowledge and conservation-relevant information. Specifically, we aimed to (i) compile and standardize mammal records from TESL to quantify species richness and media exposure; (ii) examine the extent to which media-derived patterns correspond to established biodiversity gradients across China and evaluate their relevance for conservation; and (iii) identify the factors associated with spatial variation in media exposure, with the goal of understanding how such programs can be improved to enhance biodiversity communication. Through this analysis, we sought to clarify both the informational value and the inherent limitations of curated mass media as a distinct component of broader efforts to integrate heterogeneous data sources into biodiversity conservation. To our knowledge, this is the first study to systematically assess curated monitoring footage from a television program as a biodiversity data source, thereby bridging the gap between systematic monitoring data and publicly accessible biodiversity records.

## 2. Materials and Methods

### 2.1. Data Sources

Mammal species names, protected area information, and recording times were primarily obtained from the subtitles of the television program TESL broadcast on CCTV (https://tv.cctv.com/lm/mjzy, accessed on 6 May 2021). Each TESL episode typically lasts 2 min and focuses on the biodiversity of a specific protected area or a thematic topic. Species conservation status was determined according to the latest IUCN Red List of Threatened Species (version 2025.2; https://www.iucnredlist.org, accessed on 15 September 2025). National conservation levels were referenced from the National Key Protected Wildlife List issued by the National Forestry and Grassland Administration, as well as the List of Terrestrial Wild Animals of Ecological, Scientific, and Social Importance. Spatial vector data for administrative boundaries were obtained from the National Basic Geographic Information Platform. Global biodiversity hotspots (version 2016.1) were downloaded from Zenodo (https://zenodo.org/records/3261807, downloaded on 29 December 2025) [25]. To our knowledge, no formal data-sharing protocol exists between protected area administrations and the TESL production team; footage is submitted on a voluntary basis.

### 2.2. Data Processing Workflow

#### 2.2.1. Species Information

Video data were extracted following a standardized workflow. Species information and protected area information shown in the videos were manually extracted through an item-by-item review of 1829 episodes of TESL by two observers, one responsible for the first-round extraction and the other for double-checking. Upon double-checking, 31 discrepancies were identified across the 1829 episodes (disagreement rate: 1.69%), primarily involving species identification. All discrepancies were resolved through joint review of the original videos and consultation of taxonomic databases. Each identification was cross-referenced with the Checklist of Chinese Mammals (2024 edition) [23]. No automated species recognition tools were used in this study. Species names and Latin names were standardized according to the Checklist of Chinese Mammals (2024 edition) [23]. In cases of discrepancies in Chinese common names or historical nomenclature, Latin names were used as the primary taxonomic reference. Based on this, we added taxonomic information (order and family), the IUCN threat category, and the corresponding protection level under China’s National Key Protected Wildlife List after the common name and scientific (Latin) name of each species.

#### 2.2.2. Spatial Information

Protected area information displayed in the videos was not always presented in a standardized format, and we therefore standardized all protected area names by referring to the National Nature Reserve Directory [26]. Each protected area name was unified using three elements: province, protection level, and reserve name. Administrative attributes (province, city, county) were assigned accordingly and all appended to each record.

China’s national park system has been established since 2019 and almost merged from several adjacent national or provincial nature reserves. However, protected areas that have already been incorporated into a national park plan were sometimes labeled as the name of the protected area and sometimes as a certain national park (or a specific section of the national park), inducing unwanted confusion and unevenly spatial scales for further analysis. We therefore unified the protected area information into the latest standard: for example, “Tangjiahe National Nature Reserve” and “Wanglang National Nature Reserve” were integrated into “Giant Panda National Park”. In addition, national parks often span multiple administrative units (two or more provinces), and the records of these protected areas which lacked county-level spatial information were excluded from potential new distribution verification. Records involving multiple provinces were split equally among provinces for analyses (e.g., considering the species occur in all adjacent provinces of the national park). All spatial analyses were therefore conducted at provincial and regional scales.

Of the 34 provincial-level administrative units in mainland China, the TESL episodes covered 29 provinces. The five provinces not covered—Hong Kong, Macau, Taiwan, Tianjin, and Shanghai—were excluded due to the lack of relevant episode records. To present a large-scale spatial pattern of species richness, provincial administration units were grouped into six regions following the standard geographic division of the National Bureau of Statistics [27] and China’s zoogeographic regionalization scheme [28]. This regional framework is consistent with biodiversity analyses in previous studies [1,29] (Table 1).

### 2.3. Analysis Methods

#### 2.3.1. Concordance Assessment

To evaluate the representativeness of media-derived data for biodiversity spatial analysis, we used Spearman’s rank correlation to assess consistency between media-derived and officially reported species richness across provinces. The species richness at the provincial scale were derived from Catalogue of Life China (2025) (http://www.sp2000.org.cn/CoLChina, accessed on 1 April 2026). Furthermore, Spearman’s rank correlation was used to examine the relationship between the number of national representative species and the number of play records using the related packages in R (version 4.4.1).

#### 2.3.2. Media Exposure Patterns

To avoid conflating media exposure with ecological abundance, we distinguished two media-derived metrics.

Media-exposed richness (ME_RI) was defined as the number of terrestrial mammal species documented at least once in The Eyes of the Secret Land (TESL) within a given province. As a presence-based richness metric, ME_RI reflects the size of the recorded species pool rather than the completeness of the true provincial species inventory.

Species accumulation curves were generated using the specaccum function in the vegan package (v. 2.7-1) in R, based on 1000 random permutations. We further applied an auxiliary threshold corresponding to 80% of the total observed species richness to evaluate documentation efficiency.

Importantly, the appearance frequency of a given species in the program is likely shaped jointly by the volume of raw records available to the production team and by editorial selection. Therefore, in the absence of concordance assessment, appearance frequency should be interpreted as a proxy for media attention rather than as an indicator of ecological abundance or occupancy probability.

#### 2.3.3. Modeling Media-Exposed Mammal Richness

Data originate from province-level mammal counts extracted from the TESL program videos, spanning six years and covering 29 of the 34 provincial-level administrative units in mainland China. The primary aim is to evaluate, within a Negative Binomial + offset framework, whether protected area indicators and regional information contribute to province-level cumulative number of observed species. The key provincial variables are Y (media exposed mammal species richness), *N* (official mammal species richness, as an offset), *episodes* (number of program episodes mentioned the province), *area* (province area), *lon* and *lat* (centroid coordinates of the province), *protect_total* (total number of protected areas in the province), *protect_area* (total protected area in the province), *protect_nat* (national-level protected areas), *protect_prov* (provincial-level protected areas), and *region* (provincial regional classification used as a fixed effect in sensitivity analyses).

Negative binomial regression with log link and offset(log(*N*)) was used to model count data with potential overdispersion [30,31]. Two models were compared: Model 1 (without *region*) and Model 2 (with *region* as a factor). Two NB + offset models are specified. The primary model, nb_model, excludes region and is defined as Y ~ *episodes* + *area* + *lon* + *lat* + *protect_total* + *protect_area* + *protect_nat* + *protect_prov* + offset(log(*N*)). The comparative model, nb_model_region, includes region as a fixed effect: Y ~ *episodes* + *area* + *lon* + *lat* + *protect_total* + *protect_area* + *protect_nat* + *protect_prov* + *region* + offset(log(*N*)).

Variance Inflation Factors (VIF, threshold = 5) assessed multicollinearity using the car package (v. 3.1-3). DHARMa simulations (*n* = 1000) tested for dispersion and zero-inflation. Moran’s I (k = 4 nearest-neighbor spatial weights matrix) evaluated spatial autocorrelation of Pearson residuals using the spdep package (v. 1.4-1) [32]. Models were compared by Akaike Information Criterion [31].

All analyses were performed in R version 4.4.1 (Hartig.2020). Packages used included: dplyr (v. 1.1.4) for data manipulation; MASS (v. 7.3-60.2) for negative binomial regression (glm.nb function); car for multicollinearity assessment (VIF); DHARMa (v. 0.4.7) for model diagnostics; ggplot2 (v. 4.0.0) for visualization; and spdep, sf (v. 1.0-21) and spData (v. 2.3.4) for spatial analysis and Moran’s I testing.

#### 2.3.4. Large-Scale Biodiversity Pattern and New Records Identification

Large-scale biodiversity patterns were analyzed at three spatial scales: regional, provincial and protected area. The values used for mapping were based on recorded mammal species richness obtained from TESL. To conduct a preliminary assessment of the relative importance of different protection levels in contributing to TESL video resources, we applied the Kruskal–Wallis H test.

Identification of new distribution records was based on species distribution mapping conducted in ArcGIS 10.8. Analyses were performed at the provincial and county levels due to limitations in the availability of protected area vector data. New distribution records were identified by comparing occurrence counties with IUCN Red List distribution vector data (February 2025 version) in ArcGIS. When an occurrence county lay outside the known distribution range, the Generate Near Table tool in ArcGIS 10.8 was used to calculate the minimum Euclidean distance between the boundary of the species’ IUCN distribution polygon and the polygon of the potential newly recorded county. Following Chen et al. [1], we adopted a more conservative criterion, applying a 50 km distance threshold to identify new distribution records.

## 3. Results

### 3.1. Media-Exposed Species Richness and Exposure Pattern

A total of 118 mammal species were documented from The Eyes of Secret Land videos between 2019 and 2024, representing eight orders and 32 families (Appendix A Table A1). Among these, 53 species were classified as national first-class protected animals and 35 species as national second-class protected animals. According to the IUCN Red List, the recorded species included five Critically Endangered (CR), 27 Endangered (EN), and 20 Vulnerable (VU) taxa.

**Figure 1:**
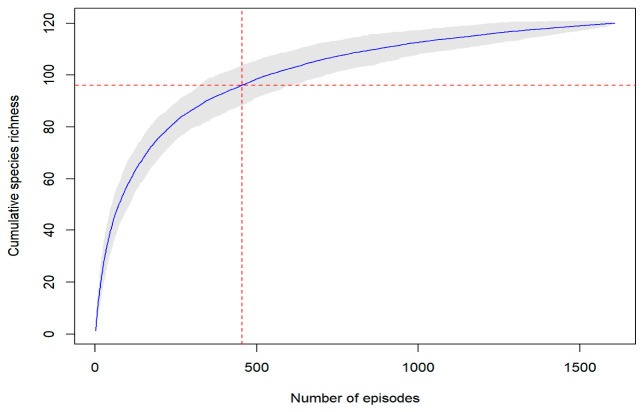
Species accumulation curve for mammals based on video records from “The Eyes of Secret Land” (2019–2024). The solid line shows the mean cumulative number of recorded species, and the gray shaded area indicates variation across 1000 random permutations.

**Figure 2:**
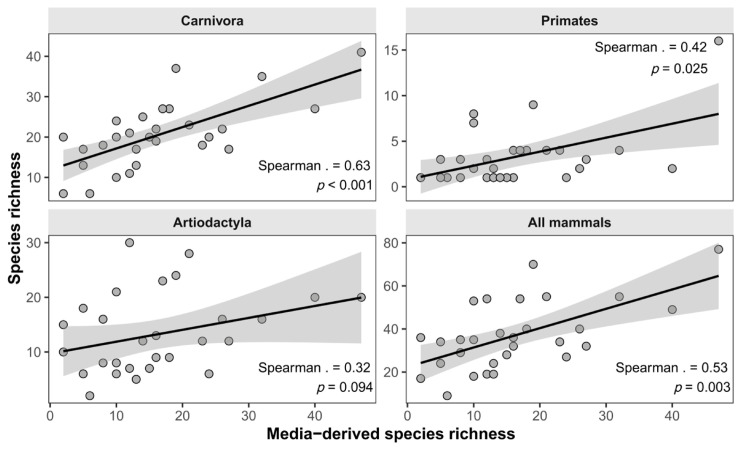
Taxon-specific relationships between media-derived species richness and official species richness at the provincial level. Each point represents one province. Solid lines indicate fitted linear trends, and gray shaded areas represent 95% confidence intervals.

The species accumulation curve exhibited a rapid initial increase (80% records within 500 episodes) followed by a clear deceleration phase, approaching an asymptote with increasing video samples (Figure 1). Species records spanned a wide range of taxonomic and ecological groups, including large ungulates, carnivores, and primates, indicating broad taxonomic coverage despite uneven observation effort (Figure 2).

### 3.2. Concordance Analysis

A correlation analysis was conducted between the species richness derived from the provincial-level data extracted from TESL videos and the provincial mammal richness from national databases. At the provincial level, media-derived species richness was positively correlated with species richness reported in the national biodiversity report (Spearman’s ρ = 0.53, *p* = 0.003; Figure 2). When all mammal species were considered together, this result indicates a moderate and statistically significant correspondence between media-derived and official richness patterns. In addition, there was a strong correlation between broadcast frequency and official species richness at province level (Spearman’s ρ = 0.51, *p* = 0.004, Figure 2).

**Figure 3:**
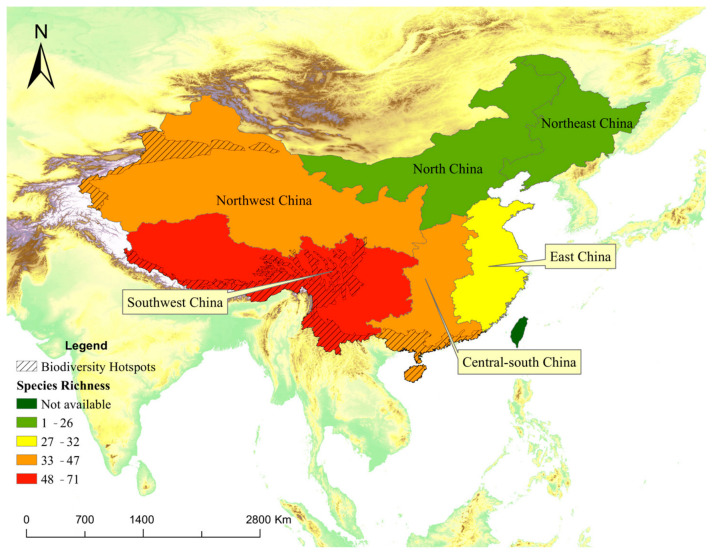
Mammal species richness spatial pattern at regional level in mainland China based on mass-media records (TESL).

Analyses conducted separately for different taxonomic orders revealed pronounced differences in correlation strength (Figure 3). The strongest relationship was observed for Carnivora, for which media-derived richness was highly correlated with official richness (ρ = 0.63, *p* < 0.001). A weaker but still statistically significant correlation was detected for primates (ρ = 0.42, *p* = 0.025). In contrast, the relationship for artiodactyla was comparatively weak and not statistically significant (ρ = 0.32, *p* = 0.094).

### 3.3. Spatial Patterns of Exposed Diversity

At the regional level (Figure 3), 71 mammal species were identified in southwest China, followed by northwest China (47), central-south China (45) and east China (32). Mammal species found in both northeast China (26) and north China (23) were less than 30.

**Figure 4:**
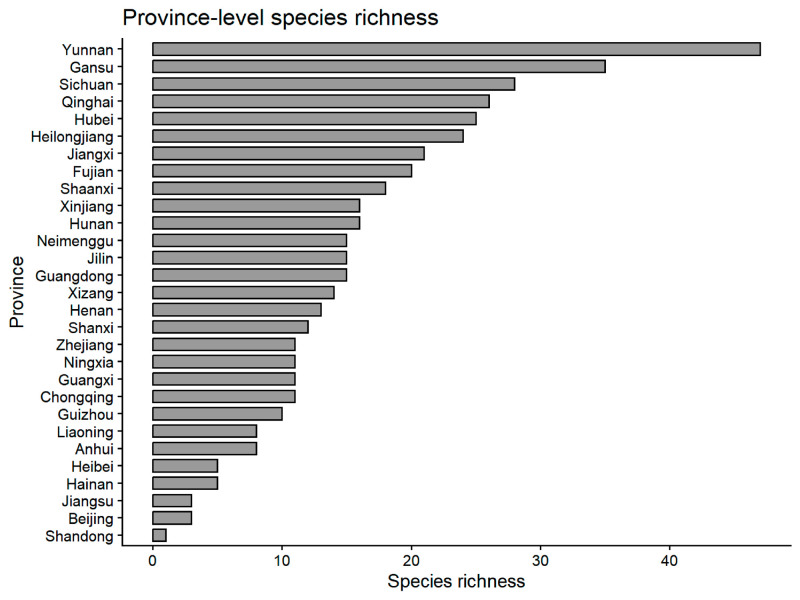
Province-level species richness of media exposure based on species-specific play counts (TESL).

**Figure 5:**
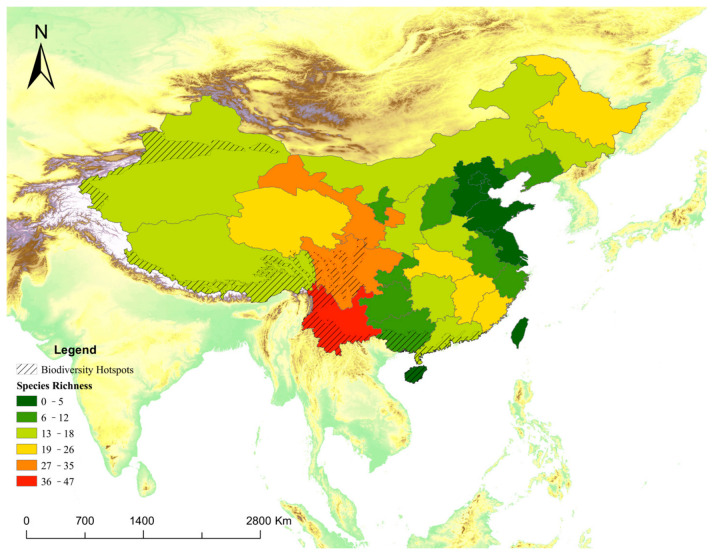
Mammal species richness spatial pattern at provincial level in mainland China based on mass-media records (TESL).

At the provincial level, provinces in southwestern China, particularly Yunnan (47), Gansu (35) and Sichuan (28), exhibit consistently high exposure-based diversity. In contrast, several provinces (Jiangsu, Beijing and Shandong) show much lower diversity values (Figure 4 and Figure 5).

At the protected area level, 285 terrestrial protected areas were featured in the program, accounting for 4.23% of the total number of the same type of protected area in China. A total of 52.5% of records came from national-level nature reserves (1249 records), which is significantly higher than other types of protected areas (Kruskal–Wallis test: χ^2^ = 32.63, *p* < 0.001). National parks contributed 25.8% of the footage, while provincial-level protected areas contributed only 0.6%.

Four national parks dominated the first five protected areas of the highest mammal species richness, including Giant Panda National Park (*n* = 26), Qilian Mountain National Park (*n* = 22), Tongbiguan Nature Reserve (*n* = 19), Shennongjia National Park (*n* = 17), and Sanjiangyuan National Park (*n* = 16).

### 3.4. Determinants of Media-Exposed Species Richness

Given the pronounced unevenness in the species exposure patterns described above, we further examined the factors associated with media-exposed species richness using generalized linear models (Appendix A Table A2). We emphasize that these models describe the determinants of media exposure—i.e., the factors influencing which species and regions appear in TESL—rather than the ecological drivers of biodiversity per se. The predictors identified below should therefore be interpreted as reflecting sampling and editorial processes rather than, or in addition to, underlying ecological gradients.

**Figure A1:**
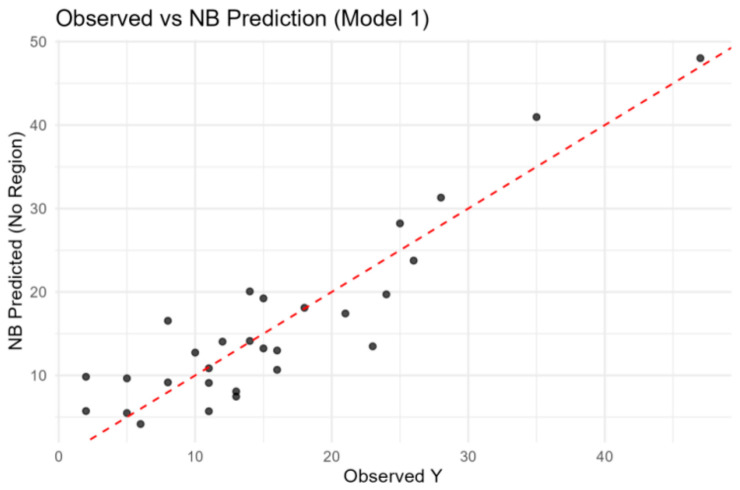
Observed Y versus NB predicted values from Model 1 (no region). The red dashed line represents y = x.

**Figure A2:**
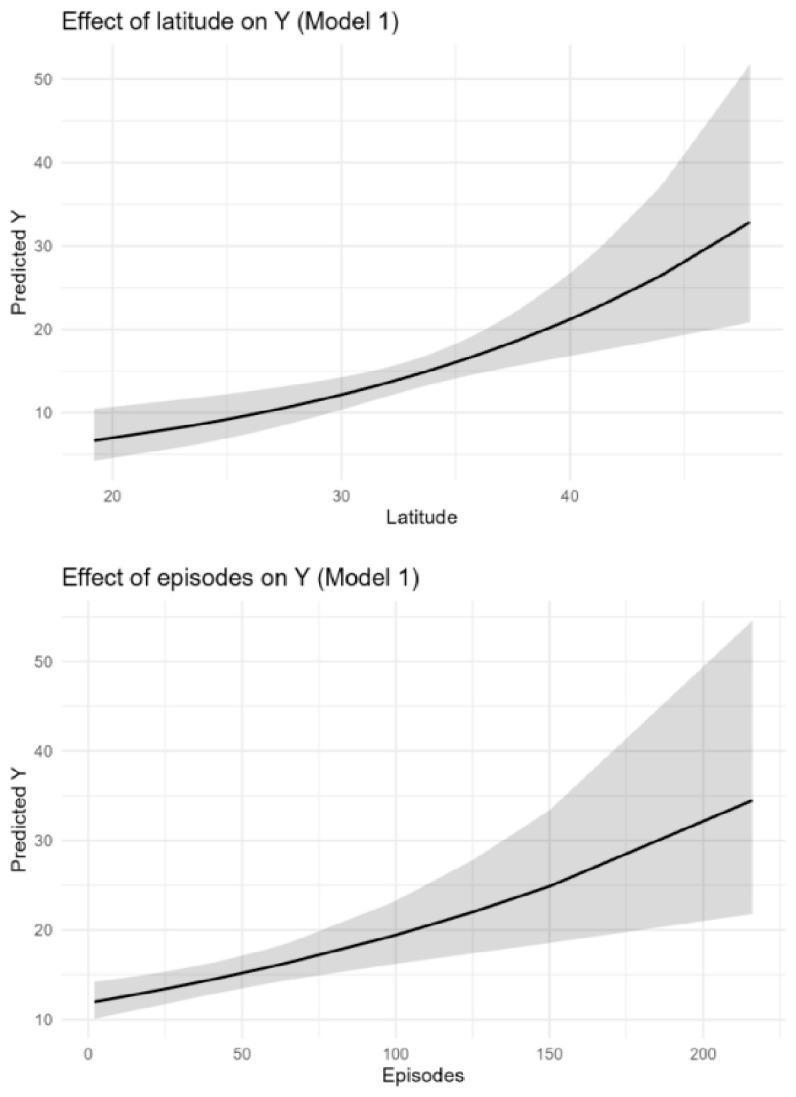
Partial effect of latitude and episodes on predicted Y (Model 1). The solid line represents the model-predicted mean response, and the gray shaded area indicates the 95% confidence interval.

There were three predictors that reached statistical significance in Model 1 (NB Regression Without Region, Figure A1 and Figure A2), including episodes: β = 0.00494 (SE = 0.00135, z = 3.66, *p* = 0.0003); area: β = −7.4 × 10^−5^ (SE = 3.10 × 10^−5^, z = −2.39, *p* = 0.017), and latitude: β = 0.0557 (SE = 0.0156, z = 3.58, *p* = 0.0003). The longitude and protection variables were not significant. Model fit: theta = 38.6, residual deviance = 37.43 (df = 20), and AIC = 194.1. Diagnostics confirmed no multicollinearity (max VIF = 4.82), appropriate dispersion (statistic = 0.798, *p* = 0.638), and no zero-inflation.

In Model 2 (NB Regression with Region), only episodes remained highly significant (β = 0.00505, SE = 0.00137, z = 3.69, *p* = 0.0002), while latitude became marginally significant (*p* = 0.096); area lost significance (*p* = 0.119). Regional indicators were not significant (all *p* > 0.05). Model fit: theta = 46.0, residual deviance = 37.46 (df = 15), and AIC = 202.83. Diagnostics were acceptable (dispersion *p* = 0.708, zero-inflation *p* = 1.0). AIC favored Model 1 (Δ AIC = 8.73).

Moran’s I test on Pearson residuals was non-significant for both Model 1 (I = 0.026, *p* = 0.286) and Model 2 (I = −0.032, *p* = 0.485), indicating no spatial autocorrelation in residuals.

**Figure A3:**
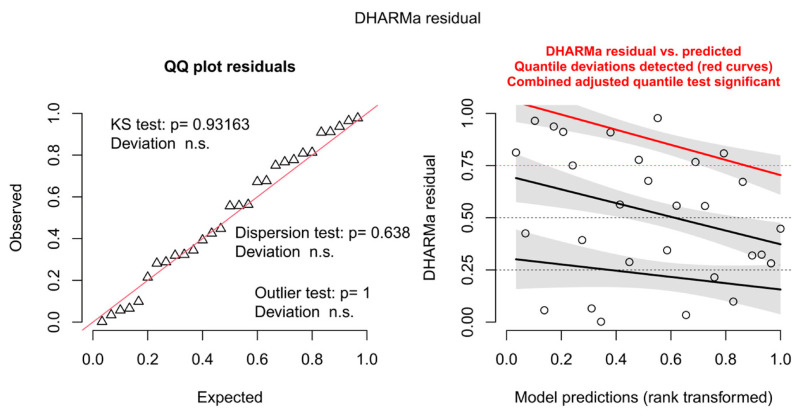
DHARMa residual diagnostics for the NB model (Model 1). (**Left**): QQ plot of residuals. Triangles indicate observations with extreme residual values relative to the expected distribution. (**Right**): DHARMa residuals vs. model predictions. Circles represent individual scaled residuals, black lines indicate the fitted quantile regression lines, grey shaded areas show the expected simu-lation envelope, and the red line indicates significant quantile deviation from model expectations. n.s.—not significant.

In summary, Model 1 without regional factors consistently outperformed Model 2 (ΔAIC = 8.73). Episodes and latitude were consistent predictors of media-exposed richness across both model specifications. However, we reiterate that these predictors reflect a mixture of sampling processes and ecological gradients: episodes is a pure sampling driver, latitude likely captures both ecological reality and monitoring bias, and the non-significance of protected area variables suggests that conservation investment alone does not guarantee media visibility. All diagnostic checks supported model validity (Figure A3, Table A1 and Table A2).

### 3.5. Identification of Potential New Distribution

**Figure 6:**
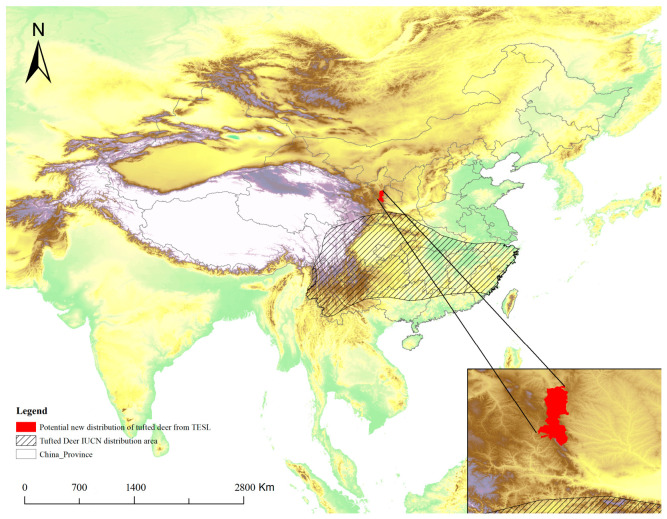
IUCN distribution area of the Tufted Deer and potential new distribution in China between 2019 and 2024 in TESL.

**Figure 7:**
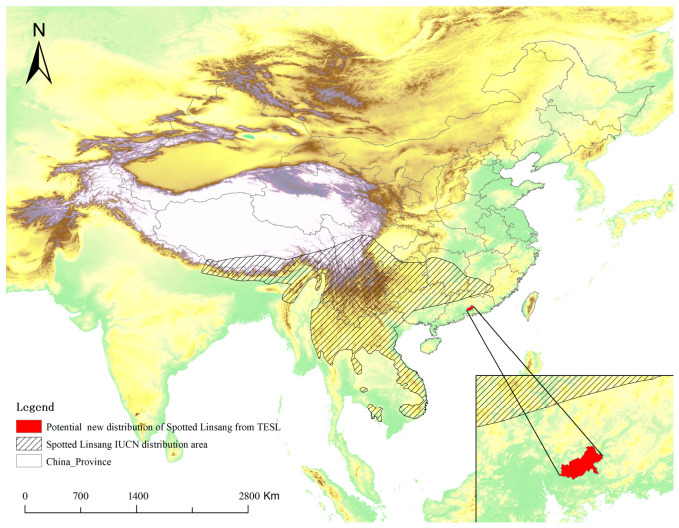
IUCN distribution area of the Spotted Linsang and potential new distribution in China between 2019 and 2024 in TESL.

**Figure 8:**
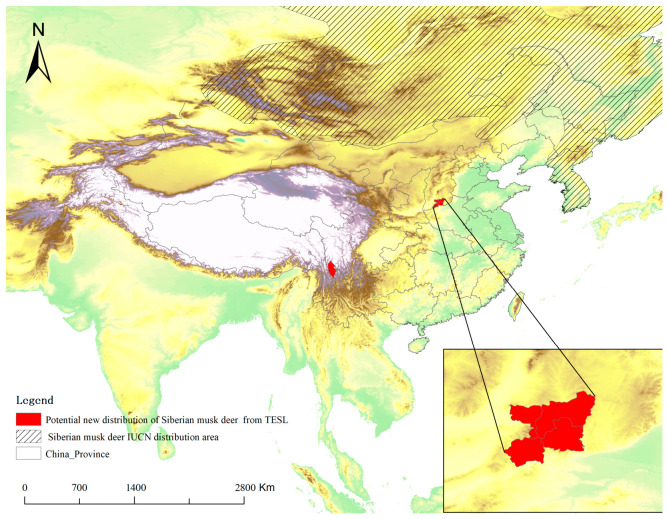
The original IUCN distribution area of the Siberian Musk Deer and the potential new distribution county in China between 2019 and 2024 in TESL.

Based on a comparison with IUCN species distribution data and the criteria for identifying new distributions, three species were identified as adding new distribution records (Table 2). These species are the Tufted Deer (*Elaphodus cephalophus*, Figure 6), the Spotted Linsang (*Prionodon pardicolor*, Figure 7), and the Siberian Musk Deer (*Moschus moschiferus*, Figure 8). All three species were recorded outside of the known IUCN distribution areas, with distances exceeding 100 km, which is much greater than the threshold for new distribution identification (50 km).

## 4. Discussion

This study shows that mammal records derived from The Eyes of the Secret Land (TESL) can recover broad-scale biodiversity patterns across mainland China despite substantial sampling bias. At the provincial scale, TESL reproduced the major richness gradient documented by official biodiversity databases, with southwestern China—particularly Yunnan and Sichuan in the Hengduan Mountains region—consistently emerging as the national hotspot of mammalian diversity [3]. However, insufficient monitoring in remote regions such as Xinjiang and Xizang may explain why known biodiversity hotspots do not fully overlap with all potential hotspot provinces. These findings suggest that professionally curated mass-media records, although incomplete and non-random, can still retain meaningful macroecological signals when analyzed at coarse spatial grains.

The ability of TESL to reflect large-scale diversity patterns is notable because the underlying data are shaped by both ecological and editorial filters. Species featured in the program are not sampled systematically; instead, their inclusion is influenced by monitoring opportunity, species detectability, and the tendency of protected area managers and editors to prioritize charismatic or emblematic mammals [33,34]. In practice, video materials are often submitted by reserve administrations to demonstrate conservation outcomes, which likely increases the representation of iconic species such as giant pandas, golden monkeys, and snow leopards. As a result, TESL does not provide an unbiased picture of community composition. Rather, it captures a selective but still informative subset of biodiversity, in which the availability of footage is likely associated with species prominence, rarity, and regional conservation visibility [35]. Furthermore, the fact that the ratio of threatened large carnivores is higher than that of threatened ungulates may explain the stronger correlation between exposed provincial species richness and official data [36]. Many ungulate species are less charismatic than large carnivores and primates, inhabit open habitats where camera-trap density is typically lower, and some of which are behaviorally cryptic, reducing their likelihood of producing broadcast-suitable footage [16,33]. These factors collectively reduce the representativeness of artiodactyl records in TESL, thereby weakening the concordance between media-exposed and official richness for this order.

Even so, the broad concordance between TESL and official datasets indicates that strong underlying geographic gradients can persist despite uneven observation from media exposure. This is consistent with previous studies showing that spatially extensive but imperfect biodiversity surrogates can still capture major diversity patterns when examined at sufficiently coarse resolutions [37,38]. In this sense, the main value of media-derived data lies not in reconstructing complete species inventories, but in preserving relative spatial differences in biodiversity. The persistence of the southwestern hotspot in our results supports the view that macroecological structure is robust enough to remain detectable even when records are filtered through institutional and media-selection processes.

Species accumulation analysis further supports this interpretation. TESL showed rapid early accumulation of species, followed by a clear deceleration as episode numbers increased. This pattern suggests that professionally produced wildlife media can efficiently capture a broad regional baseline of conspicuous mammals, but quickly approaches saturation because the most detectable and widely recognized species are documented first [39]. The fact that approximately 80% of species could be accumulated within 500 episodes indicates substantial efficiency in biodiversity representation, while the high coverage of nationally protected mammals—over 60% for first-class species and 95% for second-class species—suggests that the program performs particularly well in representing conservation-relevant taxa. Together, these results imply that TESL is especially effective at communicating species richness, rarity, and conservation significance to the public.

At the same time, the media-derived species richness patterns are clearly influenced by uneven exposure. Our Negative Binomial GLM showed that accumulated provincial richness was associated with sampling effort, province area, latitude, and regional broadcasting intensity. However, these effects should not be interpreted as purely ecological. Likewise, the weak relationship between accumulated richness and protected area size or number probably results from the selective use of footage, rather than indicating that reserve coverage has little relevance to mammal diversity [40,41]. These findings suggested a possible way to improve the performance of national or provincial typical biodiversity publicity. In practice, the producer or the consult team of the program could arrange the exposure intensity/frequency refer to the official data and adopt stratify sampling framework to enhance the evenness of exposure at provincial scale. This dual character defines both the strength and the limitation of media-derived biodiversity records. Their strength is that they can reveal broad patterns, identify major diversity centers, and potentially highlight overlooked distributional information [13]. For instance, records of tufted deer in Liupan Mountain National Nature Reserve and Siberian musk deer in Shanxi Province may point to potentially important distribution updates or remnant populations. However, such records should be treated as hypothesis-generating observations rather than definitive evidence of range shifts or local community structure [42]. Their main scientific value lies in guiding further verification, targeted surveys, and follow-up monitoring.

These findings also have practical conservation implications. In many protected areas, especially remote or less well-known ones, monitoring data remain underused in academic publication and are often inaccessible to external researchers. Media products based on such monitoring therefore contain biodiversity information that is rarely mobilized for scientific inference. Converting these records into analyzable data offers a useful supplementary pathway for biodiversity assessment and may help fill gaps in formal databases. This is particularly relevant because previous studies have noted that the accuracy of species distribution assessments, including those used by the IUCN, remains limited for many poorly studied taxa [43]. Exploring unconventional but traceable data sources may therefore improve the timeliness and completeness of conservation-relevant information.

Nevertheless, several limitations must be emphasized. TESL records are inherently non-random, taxonomically biased, and spatially imprecise. They are most informative for medium- to large-bodied, easily detected mammals and for analyses conducted at broad spatial scales. By contrast, they are much less suitable for assessing cryptic or small-bodied species, estimating abundance, or conducting fine-scale habitat analyses without independent validation [44,45]. Accordingly, media-derived biodiversity data should be regarded as complementary rather than substitutive.

## 5. Conclusions

Overall, this study demonstrates that long-term, expert-curated mass-media programs can serve as a meaningful supplementary source for macroecological biodiversity research. Although TESL does not provide complete or unbiased species inventories, it preserves large-scale diversity structure across China and captures a substantial proportion of conservation-priority mammals. More broadly, these results suggest that traditional mass media—when interpreted within its institutional, editorial, and ecological context—represents an underused but promising component of an expanded biodiversity information framework. At the same time, such analyses can feed back into program design by encouraging broader geographic representation, greater taxonomic balance, and more systematic biodiversity communication.

## Figures and Tables

**Table 1:** Regional division standards for TESL data analysis.

Region	Provinces
Northwest	Shaanxi, Gansu, Qinghai, Ningxia, Xinjiang
Southwest	Sichuan, Yunnan, Guizhou, Tibet, Chongqing
Northeast	Liaoning, Jilin, Heilongjiang
Central South	Henan, Hubei, Hunan, Guangdong, Guangxi, Hainan
North China	Beijing, Tianjin, Hebei, Shanxi, Inner Mongolia
East China	Shanghai, Jiangsu, Zhejiang, Anhui, Fujian, Jiangxi, Shandong

**Table 2:** New distribution records based on data extracted from TESL (2019–2024).

Species	National * Protected Level	IUCN Red List Category	New Record County	Distance to the Edge of IUCN Polygon
Siberian Musk Deer *Moschus moschiferus*	I	EN	Yicheng, Huanqu and Hecheng county of Shanxi province; Zhongdian county of Yunnan province	>800 km/>1500 km
Tufted Deer *Elaphodusc ephalophus*	II	NT	Jingyuan, Yuanzhou and Longde county of Ningxia Hui Autonomous Region	>130 km
Spotted Linsang *Prionodon pardicolor*	II	LC	Boluo County of Guangdong province	>100 km

* Protection class: I. National first-class protected animal; II. National second-class protected animal.

## Data Availability

The data underlying this study have been deposited in the Dryad Digital Repository (DOI: https://doi.org/10.5061/dryad.pc866t244) and will be made publicly available following completion of Dryad’s curation process.

## Appendix A

**Table A1:** 2019–2024 Catalog of Mammalian Species from TESL.

Species	National Protected Category	IUCN Red List	Number of Recorded Protected Areas	Number of Recorded Provinces
CETARTIODACTYLA				
Bovidae				
Gaur *Bos gaurus*	I	VU	1	1
Wild Yark *Bos mutus*	I	VU	6	4
Burmese Goral *Naemorhedus evansi*	II	VU	1	1
Siberian Ibex *Capra sibirica*	II	NT	4	2
Chinese Serow *Capricornis milneedwardsii*	II	VU	46	15
Long-tailed Goral *Naemorhedus caudatus*	II	VU	1	1
Chinese Takin *Budorcas tibetana*	I	VU	21	4
Himalayan Serow *Capricornis thar*	I	VU	1	1
Goitered Gazelle *Gazella subgutturosa*	II	VU	4	3
Himalayan Tahr *Hemitragus jemlahicus*	I	NT	1	1
Argali *Ovis ammon*	II	NT	3	2
Chinese Goral *Naemorhedus griseus*	II	EN	36	10
Tibetan Antelope *Pantholops hodgsonii*	I	NT	3	3
Bharal *Pseudois nayaur*	II	LC	18	7
Tibetan Gazelle *Procapra picticaudata*	I	EN	2	2
Przewalski ’s Gazelle Procapra przewalskii	I	EN	1	1
Himalayan Takin Budorcas taxicolor	I	EN	1	1
Cervidae				
Reeves ’Muntjac *Muntiacus reevesi*	III	LC	74	18
Siberian Roe Deer *Capreolus pygargus*	III	LC	41	15
Moose *Alces alces*	I	LC	10	2
Red Muntjac *Muntiacus vaginalis*	III	LC	16	5
Red Deer *Cervus elaphus*	II	LC	15	7
Sika Deer *Cervus nippon*	I	LC	14	7
Père David ’s Deer *Elaphurus davidianus*	I	EN	4	3
Tufted Deer *Elaphodus cephalophus*	II	NT	40	11
Black Muntjac *Muntiacus crinifrons*	I	VU	15	4
Gongshan Muntjac *Muntiacus gongshanensis*	II	DD	3	1
Chinese Water Deer *Hydropotes inermis*	II	LC	2	2
White-lipped Deer *Przewalskium albirostris*	I	VU	8	3
Sambar Deer *Rusa unicolor*	II	VU	9	5
Moschidae				
Forest Musk Deer *Moschus berezovskii*	I	EN	11	7
Alpine Musk Deer *Moschus chrysogaster*	I	EN	4	3
Siberian Musk Deer *Moschus moschiferus*	I	EN	8	5
Anhui Musk Deer *Moschus anhuiensis*	I	EN	1	1
Suidae				
Wild Boar *Sus scrofa*		LC	61	23
Tragulidae				
Lesser Mouse Deer *Tragulus kanchil*	I	LC	1	1
Camelidae				
Bactrian Camel *Camelus ferus*	I	CR	3	1
Phocoenidae				
Yangtze Finless Porpoise *Neophocaena asiaeorientalis*	I	EN	1	1
CARNIVORA				
Felidae				
Asiatic Golden Cat *Pardofelis temminckii*	I	VU	3	3
Chinese Mountain Cat *Felis bieti*	I	VU	9	3
Eurasian Lynx *Lynx lynx*	II	LC	26	8
Clouded Leopard *Neofelis nebulosa*	I	EN	4	3
Leopard *Panthera pardus*	I	EN	20	9
Pallas ’s Cat *Otocolobus manul*	II	LC	10	5
Tiger *Panthera tigris*	I	EN	9	4
Snow Leopard *Panthera uncia*	I	EN	28	6
Marbled Cat *Pardofelis marmorata*	II	VU	3	2
Leopard Cat *Prionailurus bengalensis*	II	LC	48	17
Mustelidae				
Hog Badger *Arctonyx collaris*	III	VU	38	12
Asian Badger *Meles leucurus*	III	LC	14	8
Wolverine *Gulo gulo*	I	LC	6	2
Sable *Martes zibellina*	I	LC	10	3
Siberian Weasel *Mustela sibirica*	III	LC	8	7
Eurasian Otter *Lutra lutra*	II	LC	1	1
Yellow-throated Marten *Martes flavigula*	II	LC	43	14
Steppe Polecat *Mustela eversmanii*	III	LC	2	1
Chinese Ferret-badger *Meles meles*	III	LC	9	3
Mountain Weasel *Mustela altaica*	III	LC	1	1
Canidae				
Gray Wolf *Canis lupus*	II	LC	11	6
Dhole *Cuon alpinus*	I	EN	6	4
Racoon Dog *Nyctereutes procyonoides*	II	LC	6	4
Tibetan Fox *Vulpes ferrilata*	II	LC	7	3
Red Fox *Vulpes vulpes*	II	LC	19	12
Viverridae				
Masked Palm Civet *Paguma larvata*	III	LC	2	2
Spotted Linsang *Prionodon pardicolor*	II	LC	5	5
Small Indian Civet *Viverricula indica*	I	LC		
Asian Palm Civet *Paradoxurus hermaphroditus*	II	LC	2	2
Ursidae				
Giant Panda *Ailuropoda melanoleuca*	I	VU		
Asiatic Black Bear *Ursus thibetanus*	II	VU	50	17
Brown Bear *Ursus arctos*	II	VU	18	9
Herpestidae				
Crab-eating Mongoose *Herpestes urva*	III	LC	13	5
Ailuridae				
Chinese Red Panda *Ailurus styani*	II	EN	9	2
Phocidae				
Spotted Seal *Phoca largha*	I	LC	3	1
RODENTIA				
Sciuridae				
Himalayan Marmot *Marmota himalayana*		LC	8	3
Maritime Striped Squirrel *Tamiops maritimus*	III	LC	6	3
Red Giant Flying Squirrel *Petaurista petaurista*	III	LC	2	2
Red-hipped Squirrel *Dremomys pyrrhomerus*	III	LC	3	3
Eurasian Red Squirrel *Sciurus vulgaris*	III	LC	2	2
Pallas ’s Squirrel *Callosciurus erythraeus*	III	LC	6	5
Pere David ’s Rock Squirrel *Sciurotamias davidianus*	III	LC	2	2
Red and White Giant Flying Squirrel *Petaurista alborufus*	III	LC	1	1
Phayre ’s Squirrel *Callosciurus phayrei*	III	LC	1	1
Siberian Chipmunk *Tamias sibiricus*	III	LC	1	1
Perny ’s Long-nosed Squirrel *Dremomys pernyi*	III	LC	1	1
Asian Red-cheeked Squirrel *Dremomys rufigenis*	III	LC	1	1
Black Giant Squirrel *Ratufa bicolor*	II	NT	4	2
Hystricidae				
Malayan Porcupine *Hystrix brachyura*	III		23	10
Castoridae				
Eurasian Beaver *Castor fiber*	I	LC	2	1
Muridae				
Spiny Taiwan Niviventer *Niviventer coninga*		LC	2	2
PRIMATES				
Cercopithecidae				
Assam Macaque *Macaca assamensis*	I	NT	7	3
North Pig-tailed Macaque *Macaca leonina*	I	VU	2	1
Stump-tailed Macaque *Macaca arctoides*	II	VU	4	1
Rhesus Macaque *Macaca mulatta*	II	LC	39	14
Tibetan Macaque *Macaca thibetana*	II	NT	22	9
Yunnan Snub-nosed Monkey *Rhinopithecus bieti*	I	EN	2	1
Guizhou Snub-nosed Monkey *Rhinopithecus brelichi*	I	CR	1	1
Sichuan Snub-nosed Monkey *Rhinopithecus roxellana*	I	EN	25	5
François’ Langur *Trachypithecus francoisi*	I	EN	7	3
White-headed Langur *Trachypithecus leucocephalus*	I	EN	2	1
Capped Langur *Trachypithecus pileatus*	I	EN	1	1
Nepal Gray Langur *Semnopithecus schistaceus*	I	LC	1	1
Shortridgei’ s Langur *Rhinopithecus strykeri*	I	EN	3	1
Indochinese Gray Langur *Trachypithecus crepusculus*	I	EN	1	1
White-cheeked Macaque *Macaca leucogenys*	II	LC	1	1
Hylobatidae				
Gaoligong Hoolock Gibbon *Hoolock tianxing*	I	EN	3	1
Hainan Gibbon *Nomascus hainanus*	I	CR	7	1
Eastern Black Crested Gibbon *Nomascus nasutus*	I	CR	1	1
Western Black Crested Gibbon *Nomascus concolor*	I	CR	1	1
LAGOMORPHA				
Leporidae				
Chinese Hare *Lepus sinensis*	III	LC	2	1
Mountain Hare *Lepus timidus*	II	LC	2	1
Manchurian Hare *Lepus mandshuricus*	III	LC	1	1
Tolai Hare *Lepus tolai*	III	LC	1	1
Ochotonidae				
Plateau Pika *Ochotona curzoniae*		LC	3	2
PERISSODACTYLA				
Equidae				
Przewalski ’s Horse *Equus ferus*	I	EN	1	1
Asiatic Wild Ass *Equus hemionus*	I	NT	3	2
Tibetan Wild Ass *Equus kiang*	I	LC	4	4
PHOLIDOTA				
Manidae				
Chinese Pangolin *Manis pentadactyla*	I	EN	1	1
PROBOSCIDEA				
Elephantidae				
Asian Elephant *Elephas maximus*	I	EN	2	1

I. National first-class protected wild animals; II. National second-class protected wild animals; III. Animals under the ‘Three Musts’ protection; EN. Endangered; VU. Vulnerable; NT. Near Threatened; LC. Least Concern; DD. Data Deficient.

**Table A2:** NB model coefficients (Model 1: without region; Model 2: with region).

Model	Term	Estimate	Std.Error	z.Value	*p*.Value	Conf.Low	Conf.High
nb_model (no region)	(Intercept)	(0.9708)	1.1320	(0.8576)	0.3911	(3.2044)	1.2347
nb_model (no region)	episodes	0.0049	0.0014	3.6584	0.0003	0.0023	0.0076
nb_model (no region)	area	(0.0001)	0.0000	(2.3858)	0.0170	(0.0001)	(0.0000)
nb_model (no region)	lon	(0.0178)	0.0121	(1.4710)	0.1413	(0.0414)	0.0061
nb_model (no region)	lat	0.0557	0.0156	3.5765	0.0003	0.0252	0.0867
nb_model (no region)	protect_total	0.0022	0.0013	1.6493	0.0991	(0.0004)	0.0049
nb_model (no region)	protect_area	0.0002	0.0002	0.8042	0.4213	(0.0002)	0.0006
nb_model (no region)	protect_nat	0.0095	0.0165	0.5729	0.5667	(0.0228)	0.0415
nb_model (no region)	protect_prov	(0.0063)	0.0068	(0.9299)	0.3524	(0.0197)	0.0069
nb_model_region (with region)	(Intercept)	(1.6113)	2.7475	(0.5864)	0.5576	(7.0015)	3.7714
nb_model_region (with region)	episodes	0.0051	0.0014	3.6917	0.0002	0.0024	0.0077
nb_model_region (with region)	area	(0.0001)	0.0000	(1.5587)	0.1191	(0.0001)	0.0000
nb_model_region (with region)	lon	(0.0105)	0.0258	(0.4078)	0.6834	(0.0613)	0.0402
nb_model_region (with region)	lat	0.0466	0.0280	1.6659	0.0957	(0.0079)	0.1019
nb_model_region (with region)	protect_total	0.0025	0.0015	1.6510	0.0987	(0.0004)	0.0054
nb_model_region (with region)	protect_area	0.0001	0.0002	0.6392	0.5227	(0.0003)	0.0006
nb_model_region (with region)	protect_nat	0.0106	0.0171	0.6188	0.5360	(0.0225)	0.0435
nb_model_region (with region)	protect_prov	(0.0085)	0.0073	(1.1768)	0.2393	(0.0227)	0.0055
nb_model_region (with region)	regionCN	0.2346	0.3360	0.6981	0.4851	(0.4274)	0.9006
nb_model_region (with region)	regionCS	0.1294	0.2436	0.5313	0.5952	(0.3400)	0.6001
nb_model_region (with region)	regionNE	0.0704	0.4123	0.1707	0.8644	(0.7486)	0.8934
nb_model_region (with region)	regionNW	0.2734	0.4430	0.6170	0.5372	(0.5982)	1.1438
nb_model_region (with region)	regionSW	0.0655	0.4049	0.1618	0.8714	(0.7181)	0.8438
